# The *Arabidopsis* Elongator Subunit ELP3 and ELP4 Confer Resistance to Bacterial Speck in Tomato

**DOI:** 10.3389/fpls.2018.01066

**Published:** 2018-07-24

**Authors:** Juliana A. Pereira, Fahong Yu, Yanping Zhang, Jeffrey B. Jones, Zhonglin Mou

**Affiliations:** ^1^Department of Plant Pathology, University of Florida, Gainesville, FL, United States; ^2^Interdisciplinary Center for Biotechnology Research, University of Florida, Gainesville, FL, United States; ^3^Department of Microbiology and Cell Science, University of Florida, Gainesville, FL, United States

**Keywords:** tomato, the Elongator complex, *AtELP4*, transgenic overexpression, disease resistance, *Pseudomonas syringae*

## Abstract

Although production of tomato (*Solanum lycopersicum*) is threatened by a number of major diseases worldwide, it has been difficult to identify effective and durable management measures against these diseases. In this study, we attempted to improve tomato disease resistance by transgenic overexpression of genes encoding the *Arabidopsis thaliana* Elongator (AtELP) complex subunits AtELP3 and AtELP4. We show that overexpression of *AtELP3* and *AtELP4* significantly enhanced resistance to tomato bacterial speck caused by the *Pseudomonas syringae* pv. *tomato* strain J4 (*Pst* J4) without clear detrimental effects on plant growth and development. Interestingly, the transgenic plants exhibited resistance to *Pst* J4 only when inoculated through foliar sprays but not through infiltration into the leaf apoplast. Although this result suggested possible involvement of stomatal immunity, we found that *Pst* J4 inoculation did not induce stomatal closure and there were no differences in stomatal apertures and conductance between the transgenic and control plants. Further RNA sequencing and real-time quantitative PCR analyses revealed a group of defense-related genes to be induced to higher levels after infection in the *AtELP4* transgenic tomato plants than in the control, suggesting that the enhanced disease resistance of the transgenic plants may be attributed to elevated induction of defense responses. Additionally, we show that the tomato genome contains single-copy genes encoding all six Elongator subunits (SlELPs), which share high identities with the AtELP proteins, and that *SlELP3* and *SlELP4* complemented the *Arabidopsis Atelp3* and *Atelp4* mutants, respectively, indicating that the function of tomato Elongator is probably conserved. Taken together, our results not only shed new light on the tomato Elongator complex, but also revealed potential candidate genes for engineering disease resistance in tomato.

## Introduction

Tomato (*Solanum lycopersicum*) fruit was once thought to be poisonous, but since being integrated as part of the human diet, its popularity and consumption have increased over the years. Tomato production has an economic impact worldwide, but it is also a costly crop to produce. It is a labor-intensive crop that requires significant amount of chemical inputs to be protected from a wide variety of pests and diseases. There are a number of diseases that affect tomatoes, including bacterial speck, which is caused by the bacterial pathogen *Pseudomonas syringae* pv. *tomato*. Bacterial speck can cause up to 75% losses in yield, if present early in the production cycle (Yunis et al., 1980). Control of the disease is primarily based on application of bactericides and sanitary measures. Pathogen-free seeds and resistant varieties carrying the resistance (*R*) gene *Pto* have been implemented to control the disease (Monroe and Sasser, 1980; Pedley and Martin, 2003). However, *P. syringae* pv. *tomato* strains have evolved to overcome the *R* gene-mediated resistance in tomato (Thapa and Coaker, 2016).

Since tomatoes are susceptible to many diseases, studies involving identification of disease resistance-related genes in model plants have increased dramatically (Piquerez et al., 2014). Currently, one strategy that is being pursued is to utilize resistance-related genes identified in *Arabidopsis* and their orthologs in other plant species (Jones et al., 2014). *Arabidopsis* is a well-established model system, with the complete genome sequenced.^1^ Furthermore, multiple *Arabidopsis* genes have been cloned, characterized, and reported to confer resistance to diseases when overexpressed in diverse crop species (Lin et al., 2004; Chan et al., 2005; Lacombe et al., 2010; Schwessinger et al., 2015; Silva et al., 2017), making *Arabidopsis* a suitable source of defense-related genes for engineering resistance in tomato.

The Elongator protein (ELP) complex is a highly conserved multitasking protein complex in eukaryotes (Otero et al., 1999; Wittschieben et al., 1999; Hawkes et al., 2002; Nelissen et al., 2010; Woloszynska et al., 2016). It consists of six subunits, including ELP1 and ELP2 (scaffolds for complex assembly), ELP3 (catalytic subunit), and an accessory complex formed by ELP4–ELP6 (Svejstrup, 2007). Elongator has been shown to be involved in several distinct cellular processes, such as exocytosis, histone modification, tRNA modification, α-tubulin acetylation, zygotic paternal DNA demethylation, and miRNA biogenesis (Hawkes et al., 2002; Huang et al., 2005; Rahl et al., 2005; Creppe et al., 2009; Okada et al., 2010; Ding and Mou, 2015; Fang et al., 2015). It has been clearly demonstrated that Elongator functions in both the nucleus and the cytoplasm (Versées et al., 2010). In the nucleus, Elongator regulates histone acetylation and DNA methylation/demethylation (Winkler et al., 2002; Lin et al., 2012; Wang et al., 2013), thus being involved in gene transcription. In the cytoplasm, it is responsible for tRNA modification, which consequently regulates protein translation (Huang et al., 2005; Esberg et al., 2006; Glatt et al., 2012).

It has been well documented that the *A. thaliana* Elongator protein (AtELP) complex plays an important role in plant immunity, likely by regulating the transcription of defense genes (Ding and Mou, 2015; Wang et al., 2015). However, whether Elongator has a similar role in plant species other than *Arabidopsis* remains to be determined. Although it has been reported that silencing of a tomato *AtELP2*-like gene, *SlELP2L*, resulted in pleiotropic phenotypes similar to those of the *Atelp* mutants, defense phenotypes of the *SlELP2L*-RNAi lines were not tested (Zhu et al., 2015). Furthermore, some of the phenotypes displayed by the *SlELP2L*-RNAi lines appear to be different from those of the *Atelp* mutants. For instance, while ethylene signaling and auxin levels are elevated in the *Atelp* mutants, both are reduced in the *SlELP2L*-RNAi lines (Nelissen et al., 2010; Zhu et al., 2015). These differences suggest that the function of Elongator in tomato might not be exactly the same as that in *Arabidopsis*. Further characterization of genes encoding the Elongator subunits in tomato will not only help in understanding the function of Elongator in plants, but may also identify new strategies for improving disease resistance in tomato.

In this study, we characterized transgenic tomato plants expressing the *Arabidopsis AtELP3* and *AtELP4* genes. We show that overexpression of *AtELP3* and *AtELP4* significantly enhanced resistance to tomato bacterial speck caused by the *P. syringae* pv. *tomato* strain J4 (*Pst* J4) without clear detrimental effects on plant growth and development. Interestingly, the enhanced resistance was detected only when plants were inoculated via foliar sprays of bacterial suspensions but not infiltration into the apoplast, suggesting possible involvement of stomatal immunity. However, *Pst* J4 inoculation did not induce stomatal closure and there were no differences in stomatal apertures and conductance between the transgenic and control plants, indicating that a defense mechanism other than stomatal immunity was activated in the transgenic plants. Indeed, further RNA sequencing (RNA-seq) revealed a group of defense-related genes that were confirmed by real-time quantitative PCR (qPCR) analysis to be induced to higher levels after infection in the *AtELP4* transgenic tomato plants than in the control, suggesting that the enhanced disease resistance of the transgenic plants may be attributed to elevated induction of defense responses. Additionally, we show that the tomato genome encodes all six Elongator subunits (SlELPs) and that the tomato *SlELP3* and *SLELP4* genes complemented the *Arabidopsis Atelp3* and *Atelp4* mutants, respectively. Thus, the tomato Elongator is most likely functional and *AtELP3, AtELP4* as well as their tomato orthologs *SlELP3* and *SlELP4* could potentially be employed in the improvement of disease resistance in tomato plants.

## Materials and Methods

### Plasmid Construction and Plant Transformation

The T-DNA plasmids (pK7WG2D, 1-*AtELP3* and pK7WG2D, 1-*AtELP4*) reported previously (Silva et al., 2017) were used to transform the tomato cultivar “Moneymaker” following an *Agrobacterium tumefaciens*-mediated genetic transformation protocol (Lin et al., 2004). The tomato genetic transformation experiment was conducted by the UNL Plant Transformation Facility^2^. For complementation of the *Arabidopsis Atelp3* and *Atelp4* mutants, the coding regions of the tomato orthologs (*SlELP3* and *SlELP4*) were amplified from “Moneymaker” cDNAs by PCR using gene specific primers (Supplementary Table S1) and cloned into the binary vector pBI1.4T (Mindrinos et al., 1994). The resulting plasmids were introduced into the *A. tumefaciens* strain GV3101(pMP90) by electroporation (Shen and Forde, 1989). The *Arabidopsis Atelp3-10* and *Atelp4*/*elo1-1* mutant alleles (Nelissen et al., 2005; Defraia et al., 2010), which are in the Columbia (Col-0) and Landsberg *erecta* ecotype backgrounds, respectively, were used for *A. tumefaciens*-mediated genetic transformation following the floral dip method (Clough and Bent, 1998).

### Identification of Single T-DNA Insertion Homozygous Transgenic Lines

The T_1_ transgenic tomato plants obtained from the UNL Plant Transformation Facility were allowed to set seeds. The T_2_ plants were subjected to PCR analysis using gene-specific primers (Supplementary Table S1) to analyze T-DNA insertion copy numbers based on the expected ratio of 3:1 for a single T-DNA insertion. The transgenic lines that showed the expected ratio for a single T-DNA insertion were kept and seeds from the individual T_2_ plants were collected separately. The T_3_ progeny plants from each individual T_2_ plants were subjected to PCR analysis to identify homozygous plants for each transgenic line. Seeds from the homozygous T_3_ plants were pooled for further analysis. For *Arabidopsis* transgenic lines, T_2_ seeds from individual T_1_ plants were plated on Murashige and Skoog (MS) medium with 50 μg/mL kanamycin to identify single T-DNA insertion lines based on the segregation ratio of the neomycin phosphotransferase II (*nptII*) gene. Homozygous plants were similarly identified in the T_3_ generation.

### Pathogen Infection and Bacterial Population Assay

To evaluate the resistance of the transgenic tomato plants to bacterial speck, a bacterial suspension of *Pst* J4, adjusted to 1 × 10^8^ colony-forming units (cfu)/mL, was sprayed on 4-week-old tomato plants in pots with a diameter of 10 cm. This inoculum is able to induce consistent levels of disease severity on tomato plants (Kozik and Sobiczewski, 2000). The plants were then immediately covered with bags and a rubber band was placed around the base of the pot to seal the bag in order to maintain high humidity for 40 h. A total of six plants per line were tested and non-transformed “Moneymaker” was included as the control. Inoculated plants were incubated in the growth chamber and maintained at 22°C under a regimen of 12 h dark and 12 h light. The disease symptoms were evaluated 6 days post-inoculation. The disease assessment consisted of the following disease scores: 0 indicates no symptom development; 1 indicates few slightly visible lesions; 2 indicates a significant number of discernible lesions; 3 indicates a higher amount of discernible necrotic and chlorotic lesions; and 4 indicates extensive necrotic and chlorotic lesions and extensive dead tissue.

For quantifying bacterial populations of *Pst* J4 in the inoculated tomato plants, leaf tissues were sampled every 6 days. Three leaf disks with an area of 1 cm^2^ were obtained from each transgenic line using a cork borer. The leaf disks were placed into glass tubes and ground in 1 mL of sterile water. The resulting suspensions were diluted by making five 10-fold dilutions. The dilutions were plated on nutrient agar medium and then the plates were incubated at 28°C for 2 days. Colonies typical of *P. syringae* pv. *tomato* were counted and the bacterial number per cm^2^ of leaf tissue was calculated.

For testing growth of the bacterial pathogen *Psm* ES4326 in *Arabidopsis*, leaves were infiltrated with a suspension of *Psm* ES4326 (OD_600_ = 0.0001) using a 1 mL needleless syringe as described previously (Defraia et al., 2010). Leaf disks were collected from eight leaves 3 days post-inoculation using a cork borer. Each leaf disk was placed into a tube containing 500 μL of 10 mM MgCl_2_ and ground with a sterile pellet pestle. The resulting suspensions were serially diluted 20-fold four times. The dilutions were then plated on Trypticase Soy Agar medium supplemented with 25 μg/mL streptomycin and incubated at 28°C for 2 days. Colonies that grew on the plates were counted and the bacterial number per leaf disk tissue was calculated.

### Stomatal Conductance Measurement

Tomato plants were sprayed with a *Pst* J4 bacterial suspension (10^8^ cfu/mL). Stomatal conductance was measured before the inoculation (time 0) and every 30 min post-inoculation using a portable photosynthesis system (LI-6800, LI-COR Biosciences^3^). The principle of the measurement is that the time required to force a certain volume of air through the plant leaf is inversely proportional to leaf stomatal conductance (Rebetzke et al., 2000). Ten fully expanded leaves per plant were used for the measurement at each time point, and the readings from the abaxial side of the leaves were recorded.

### RNA Sequencing and Real-Time Quantitative PCR Analysis

Tomato plants were sprayed with a *Pst* J4 suspension (10^8^ cfu/mL). Three replicates of leaf tissues from six plants per genotype were collected at 0, 8, and 24 h post-inoculation. Total RNA was extracted from the collected leaf tissues using the RNeasy plant mini kit following the manufacturer’s protocol (Qiagen^4^). RNA concentration and quality were determined using a Qubit 2.0 Fluorometer (ThermoFisher^5^) and an Agilent 2100 Bioanalyzer (Agilent Technologies, Inc.^6^), respectively. Total RNA samples with 28S/18S >1 and RNA integrity number ≥7 were used for RNA-seq analysis. The RNA samples from the three biological replicates were pooled and equal amounts of RNA from the pooled samples were used for RNA-seq library preparation. Briefly, 1 μg of total RNA together with 2 μL of 1:200 diluted ERCC (External RNA Controls Consortium) RNA spike-in mix was used for mRNA extraction with 15 μL of NEBNext Magnetic Oligo d(T)_25_ and fragmented in NEBNext First Strand Synthesis Buffer by heating at 94°C for 8 min, then followed by first strand cDNA synthesis using reverse transcriptase and random primers. Synthesis of double-stranded cDNA was done using the second strand master mix provided in the kit. The resulting double-stranded cDNA was subjected to end-repair and dA-tailing and then ligated with NEBNext adaptors. Finally, the library was enriched by PCR amplification and purified by Agencourt AMPure beads (Beckman Coulter^7^). Barcoded libraries were sized and quantitated. qPCR was used to validate the library’s functionality, using the KAPA library quantification kit (Kapa Biosystems^8^). The six individual samples were pooled equimolarly for one lane of HiSeq 3000 2 × 100 cycles run. Sequencing was performed on the Illumina HiSeq 3000 instrument at the University of Florida Interdisciplinary Center for Biotechnology (UF ICBR) NextGen DNA Sequencing core. The reads that passed Illumina quality control filtering were cleaned up with the Cutadapt program (Martin, 2011) to trim off sequencing adaptors and low quality bases with a quality Phred-like score <20. Reads <40 bases were excluded from RNA-seq analysis. The transcripts of tomato from National Center for Biotechnology Information (NCBI) were used as reference sequences for RNA-seq analysis. The cleaned reads of each sample were mapped independently to the reference sequences using the mapper of bowtie2 with a maximum of three mismatches for each read. The mapping results were processed with the samtools and scripts developed in house at the UF ICBR to remove potential PCR duplicates and select uniquely mapping reads for gene expression estimation. The number of mapped reads for each individual gene was counted. Comparison was made between the *AtELP4* transgenic line 61-5 and the control samples collected at the same time point.

For qPCR analysis, total RNA was extracted from tomato plants using Trizol reagent (Thermo Fisher Scientific) and treated with RNAse-free DNAse I (Thermo Fisher Scientific). First strand complementary DNA was synthetized using 10 μg of total RNA with oligo (dT) primer and Moloney murine leukemia virus reverse transcriptase (Thermo Fisher Scientific). Gene-specific primers used for qPCR analysis were listed in Supplementary Table S1. qPCR was performed using ABsolute SYBR Green PCR master mix (Thermo Fisher Scientific) using the SYBR Green protocol (Applied Biosystems^9^). Reactions were run and analyzed on a MX3000P qPCR system (Agilent^10^). The relative mRNA levels of the target genes were expressed relative to the tomato *Actin* gene (Zhou et al., 2014a,b), and calculated using the ΔC*_T_* method (Wittwer et al., 2001).

### Statistical Analysis

Statistical analyses were performed using one-way ANOVA followed by a Tukey’s multiple comparisons test in Prism 7 (GraphPad Software^11^).

### Accession Number

Sequence data from this article can be found in the Arabidopsis Genome Initiative, the Tomato Genome Sequencing Project, or GenBank/EMBL databases under the following accession numbers: AtELP3 (At5g50320); AtELP4 (At3g11220); PR1b1 (Y08804.1); PR-5x (AY093595); DES (AF317515); ER1 (J04099.1); SlELP1 (Solyc05g054630); SlELP2 (Solyc06g008310); SlELP3 (Solyc03g110910); SlELP4 (Solyc11g010950); SlELP5 (Solyc02g086100); SlELP6 (Solyc12g009500); and NCBI Gene Expression Omnibus Series number GSE97697 (RNA-seq data).

## Results

### Generation and Morphological Characterization of Transgenic Tomato Lines Overexpressing *AtELP3* and *AtELP4*

**FIGURE 1:**
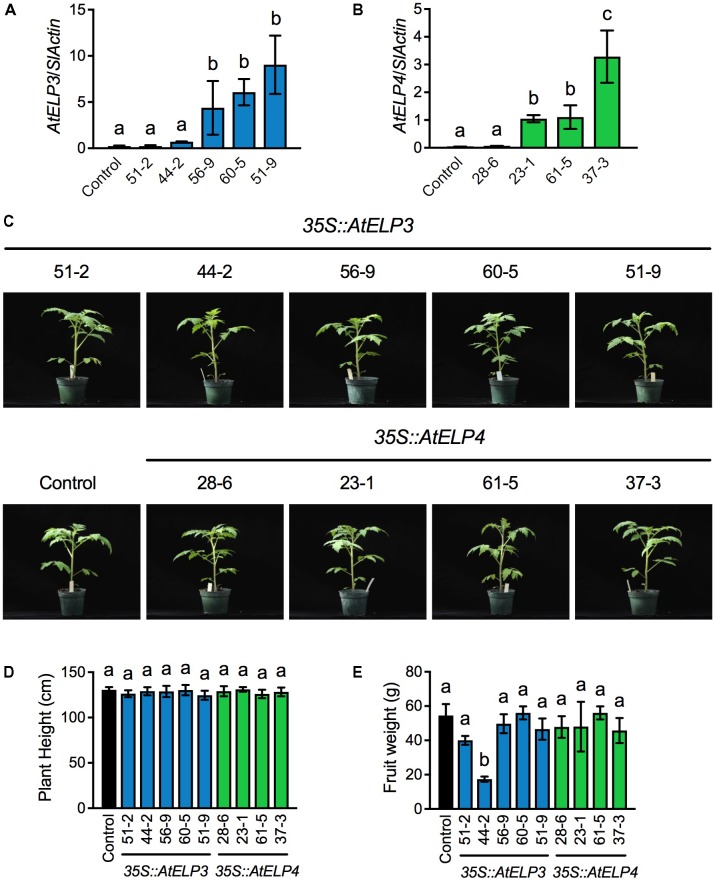
Molecular and morphological characterization of transgenic tomato lines expressing *AtELP3* and *AtELP4*. **(A,B)** Expression levels of *AtELP3* **(A)** and *AtELP4* **(B)** in independent *AtELP3* and *AtELP4* transgenic lines, respectively. Expression of the transgene was normalized against the constitutively expressed *SlActin* gene. Almost no *AtELP3* and *AtELP4* expression was detected in the non-transformed control. Data represent the mean of three biological replicates with SD. Different letters above the bars indicate significant differences (Tukey’s test, *P* < 0.05). **(C)** Morphological phenotypes of the transgenic tomato lines and the control. Photos were taken 30 days after germination. **(D)** Plant height of the transgenic lines and the control. Data represent the average of six plants with SD. Different letters above the bars indicate significant differences (Tukey’s test, *P* < 0.05). **(E)** Fruit weight of the transgenic lines and the control. Data represent the average weight of fruit from six plants with SD. Different letters above the bars indicate significant differences (Tukey’s test, *P* < 0.05).

Based on PCR amplification of the cDNA of *AtELP3* or *AtELP4* using gene specific primers (Supplementary Table S1), out of 80 T_1_ putative transgenic plants produced by the University of Nebraska–Lincoln (UNL) Plant Transformation Facility, 36 carried the *AtELP3* transgene and 35 the *AtELP4* gene. Note that the PCR reactions did not amplify any products from the non-transformed “Moneymaker” plants, which were used as the negative control in the experiment, demonstrating the specificity of the primers. The transgenic plants with T-DNA insertion were kept for seed production. Single T-DNA insertion lines were identified in the T_2_ generation based on the 3:1 segregation ratio expected for a single T-DNA insertion, and homozygosity of the single insertion lines was determined in the T_3_ generation. In total, five and four single insertion homozygous lines were identified for *AtELP3* and *AtELP4*, respectively. Expression levels of *AtELP3* and *AtELP4* in the single insertion homozygous lines were determined in the T_4_ generation by qPCR using gene-specific primers (Supplementary Table S1). While *AtELP3* and *AtELP4* transcripts were barely detectable in the control plants, the transgenes were expressed at varied levels in the different lines (**Figures 1A,B**). For *AtELP3*, expression levels of the transgene were significantly higher in lines 56-9, 60-5, and 51-9 than in lines 51-2, 44-2, and the control plants (**Figure 1A**). For *AtELP4*, the transgenic lines could clearly be classified into three groups: one high expresser (line 37-3), two medium expressers (lines 23-1 and 61-5), and one low expresser (line 28-6) (**Figure 1B**).

The overall morphology and development of the transgenic tomato plants were very similar to those of the control plants under standard greenhouse conditions (**Figure 1C**). There were no significant differences in plant height between the transgenic lines and the control (**Figure 1D**). Furthermore, all of the transgenic lines formed flowers and fruits. The fruit weight of all the transgenic lines except 44-2 was not significantly different from that of the control (**Figure 1E**). The fruit from the transgenic line 44-2 was very small and similar to that produced by cherry tomato varieties. The small-fruit phenotype of line 44-2 was unlikely caused by overexpression of *AtELP3*, since other lines (56-9, 60-5, and 51-9) that expressed higher levels of *AtELP3* than line 44-2 did not show such a phenotype. It might be possible that the small-fruit phenotype was caused by a T-DNA insertion mutation. Alternatively, there might be seed contamination during the development of transgenic plants. Nevertheless, these results indicate that transgenic overexpression of *AtELP3* and *AtELP4* does not affect tomato plant growth and development.

### Disease Resistance of the Transgenic Tomato Lines

**FIGURE 2:**
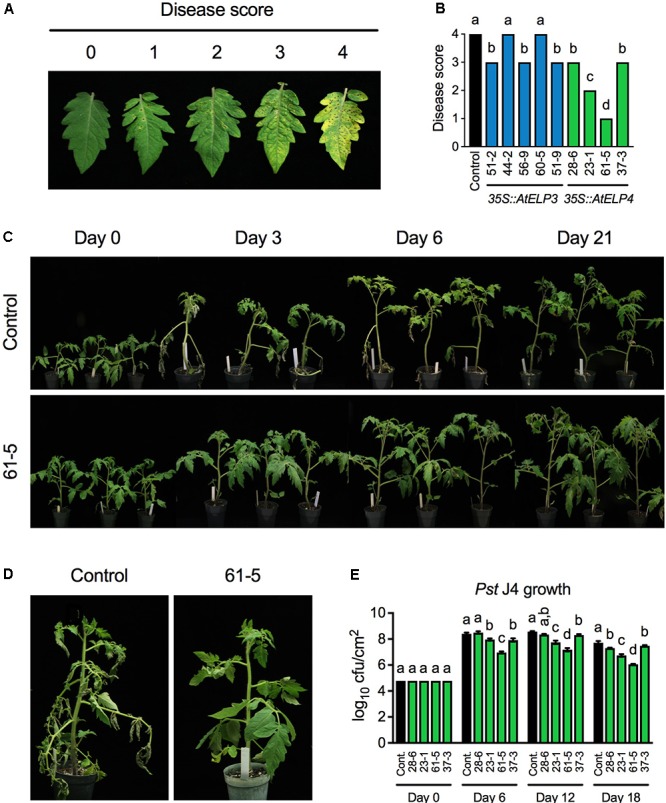
Resistance of the transgenic lines to bacterial speck disease caused by *Pst* J4. **(A)** Responses on the leaves to which different disease scores were assigned after spray inoculation with *Pst* J4. Score 0: no symptoms; Score 1: few slightly visible lesions; Score 2: significant number of discernible lesions; Score 3: higher amount of discernible necrotic and chlorotic lesions; and Score 4: extensive necrotic and chlorotic lesions and extensive dead tissue. **(B)** Disease scores of bacterial speck disease on five *AtELP3* transgenic lines, four *AtELP4* transgenic lines, and the control. Data represent the mean of six plants with SD. The disease symptoms on each line were highly uniform and the SD values were 0. Different letters above the bars indicate significant differences (Tukey’s test, *P* < 0.05). **(C)** Bacterial speck disease progression on the most resistant transgenic line 61-5 and the control following spray inoculation with *Pst* J4. **(D)** Close-up pictures of the most resistant transgenic line 61-5 and the control at 3 days post-inoculation. **(E)** Bacterial titers in the *AtELP4* transgenic lines and the control. cfu, colony forming unit. Data represent the mean of three biological replicates with SD. Different letters above the bars indicate significant differences (Tukey’s test, *P* < 0.05). Note that the comparison was made separately among the transgenic lines as well the control (cont.) for each time point.

To test whether transgenic overexpression of *AtELP3* or *AtELP4* in tomato improves disease resistance, we inoculated the single insertion homozygous transgenic lines with the bacterial pathogen *Pst* J4, which causes bacterial speck on tomato plants. Both leaf infiltration and foliar sprays were employed in the experiment, since the transgenic plants might respond differently to these two commonly used inoculation methods. The bacterial speck disease symptoms, characterized by small, black, or brown necrotic lesions surrounded by a chlorotic halo, appeared 3 days post-inoculation on the transgenic plants and the control for both inoculation methods. When the plants were inoculated using the leaf infiltration method, no significant differences were observed between the bacterial speck disease symptoms developed on any of the transgenic lines and the control, and *Pst* J4 grew to similar levels in all the tested plants (Supplementary Figure S1). In contrast, when foliar sprays were used, the disease symptoms on different transgenic lines and the control differed drastically. To quantify the disease symptoms, different disease scores were assigned to the transgenic lines based on the disease severity on the leaves (**Figures 2A,B**). The disease symptoms on the *AtELP3* transgenic lines 44-2 and 60-5 were similar to those on the control, the disease symptoms on the *AtELP3* transgenic lines 51-2, 56-9, and 51-9 as well as the *AtELP4* transgenic lines 28-6 and 37-3 were slightly less severe than those on the control, and the disease symptoms on the *AtELP4* transgenic lines 23-1 and 61-5 were much less severe than those on the control (**Figure 2B**). Interestingly, there was no clear correlation between the disease severity and the expression levels of the transgenes (**Figures 1A,B, 2B**), which is not without precedent (Luhua et al., 2008). The transgenic line 61-5, which was a medium expresser of *AtELP4* (**Figure 1B**), exhibited the strongest resistance to *Pst* J4 (**Figure 2**). The bacterial speck disease progression on the transgenic line 61-5 was markedly slower than that on the control plants (**Figure 2C**). At 3 days post-inoculation, leaves on the control had already wilted, whereas those on the transgenic line 61-5 still stayed uncurled (**Figure 2D**). We also determined bacterial titers in the *AtELP4* transgenic lines, since, based on disease symptoms, two independent *AtELP4* transgenic lines (23-1 and 61-5) displayed clear resistance to *Pst* J4. Consistent with the observed disease symptoms, the bacterial titers in the transgenic lines 61-5 and 23-1 were the lowest and the second lowest, respectively, among the *AtELP4* transgenic lines as well as the control (**Figure 2E**). These results indicate that overexpression of the *AtELP4* gene in tomato is able to significantly improve resistance to *Pst* J4-caused bacterial speck disease.

### Stomatal Conductance of the Transgenic Tomato Line With Increased Disease Resistance

**FIGURE 3:**
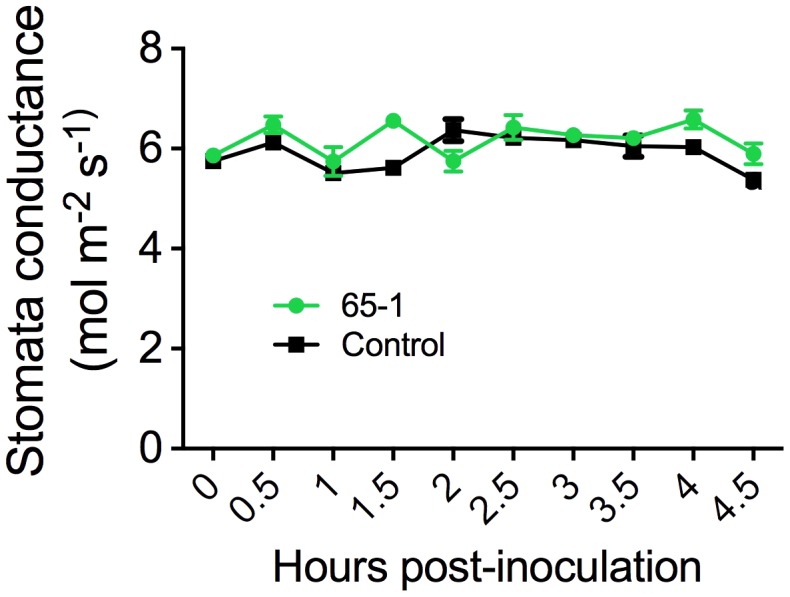
Stomatal conductance of the transgenic line 61-5 and the control. The assessment was done every 30 min after spray inoculation with *Pst* J4. Data represent the mean of five biological replicates with SD.

The different responses of the transgenic lines to leaf infiltration and foliar sprays suggested a possible involvement of stomatal immunity (Melotto et al., 2006). To test this possibility, we assessed stomatal morphology and movement after foliar sprays of *Pst* J4 under optical microscope. Interestingly, inoculation of tomato plants with *Pst* J4 did not induce stomatal closure and no appreciable differences in stomatal apertures between the most resistant transgenic line 65-1 and the control were observed (Supplementary Figure S2). We further measured stomatal conductance using a portable photosynthesis system. Overall, there were no clear differences in stomatal conductance between the transgenic line 61-5 and the control in a period of 4.5 h following foliar sprays of *Pst* J4 (**Figure 3**). Taken together, these results suggest that alteration of stomatal immunity may not be a predominant factor for the enhanced disease resistance observed in the *AtELP4* transgenic plants.

### Induction of Defense Genes in the Transgenic Tomato Line With Increased Disease Resistance

**FIGURE 4:**
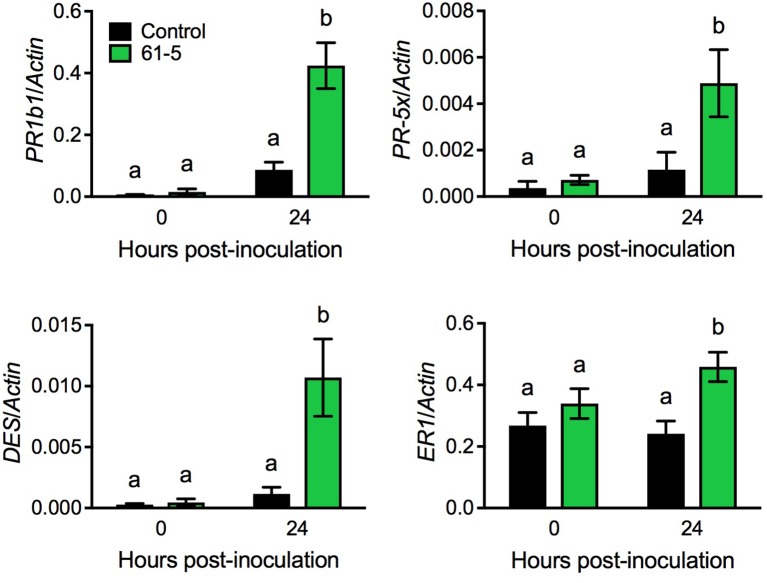
*Pst* J4-induced expression of *PR1b1, PR-5x, DES*, and *ER1* in the transgenic line 61-5. Plants were spray inoculated with the bacterial pathogen *Pst* J4 (10^8^ cfu/mL). Leaf tissues were collected at the indicated time points. Total RNA was extracted from the inoculated leaves and analyzed for the expression of the indicated genes using qPCR. Expression was normalized against the constitutively expressed *Actin* gene. Data represent the mean of three biological replicates with SD. Different letters above the bars indicate significant differences (Tukey’s test, *P* < 0.05).

To uncover potential mechanisms underlying the enhanced disease resistance of the transgenic tomato plants overexpressing *AtELP4*, we performed an RNA-seq experiment to compare *Pst* J4-induced transcriptome changes in the transgenic line 61-5 and the control, and then examined genes that were potentially induced to higher levels in the transgenic line 61-5 than in the control (NCBI Gene Expression Omnibus Series number GSE97697). Interestingly, a group of defense-related genes, including *PATHOGENESIS-RELATED* (*PR*) gene *PR1b1, PR-5* family member *PR-5x, DIVINYL ETHER SYNTHASE* (*DES*), and *ETHYLENE-RESPONSIVE* (*ER*) *PROTEASE INHIBITOR 1* (*ER1*) (GenBank accession numbers: Y08804.1, AY093595, AF317515, and J04099.1, respectively), which have previously been shown to be associated with disease resistance in tomato (Pautot et al., 1991; Ishihara et al., 2012), were potentially induced to higher levels in the transgenic line 61-5 than in the control. Since the RNA-seq experiment did not include biological replicates, statistical significance could not be evaluated. To confirm the RNA-seq results for the selected genes, we used qPCR to monitor the induction of *PR1b1, PR-5x, DES*, and *ER1* in the transgenic line 61-5 and the control after *Pst* J4 infection. Consistent with the RNA-seq results, the induction of the four selected genes in the transgenic line 61-5 was significantly higher than that in the control plants (**Figure 4**). These results suggest that the enhanced disease resistance of the transgenic plants overexpressing *AtELP4* may be due to increased induction of defense genes.

### Tomato Orthologs Encoding the Elongator Subunits

It is generally believed that the Elongator complex is highly conserved in eukaryotes. In agreement with this belief, Zhu et al. (2015) have shown that silencing of a tomato *AtELP2*-like gene, *SlELP2L*, resulted in pleiotropic phenotypes similar to those of *Atelp* mutants. Thus, the tomato genome should encode all SlELPs. To test this, BLAST searches were conducted on the tomato (*S. lycopersicum*) genome^12^ using AtELP protein sequences as the queries. The results showed that each subunit of the Elongator complex is encoded by a single-copy gene in the tomato genome. Amino acid sequence alignments indicated that the SlELP proteins all share high identities (>53%) and similarities (>70%) with the AtELP proteins (Supplementary Figure S3). Particularly, SlELP3 has very high amino acid sequence identities (93%) and similarities (96%) with AtELP3, indicating that the Elongator complex catalytic subunits are highly conserved in tomato and *Arabidopsis*. Therefore, tomato probably has a functional Elongator complex.

### Complementation of the *Atelp3* and *Atelp4* Mutants With the Tomato Orthologs

**FIGURE 5:**
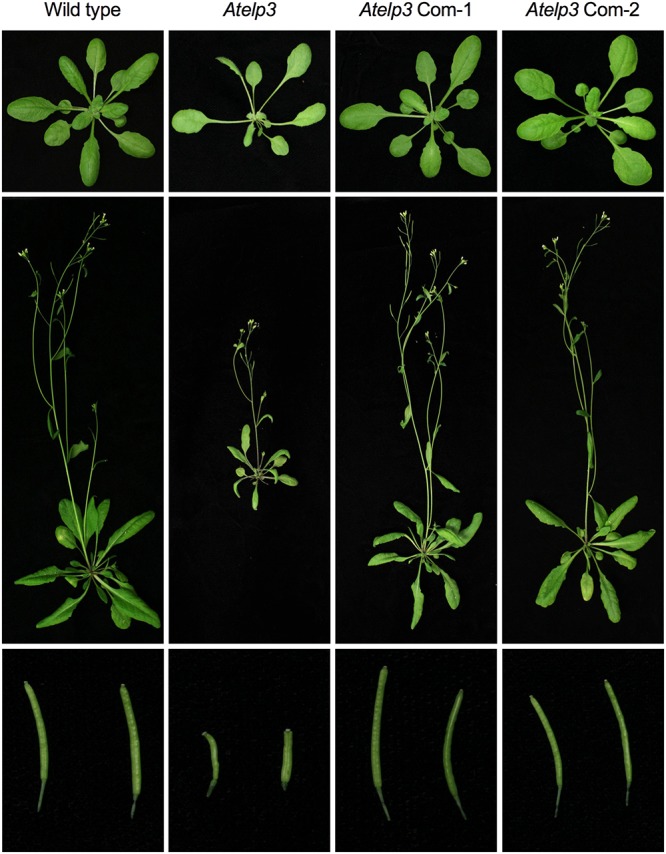
Complementation of the Arabidopsis *Atelp3* mutant by *SlELP3*. Morphological phenotypes of Arabidopsis wild type, *Atelp3*, and two independent complementation lines (*35S::SlELP3 Atelp3*): *Atelp3* Com-1 and *Atelp3* Com-2. **(Top)** 3-weak-old plants; **(Middle)** 6-week-old plants; **(Bottom)** siliques.

**FIGURE 6:**
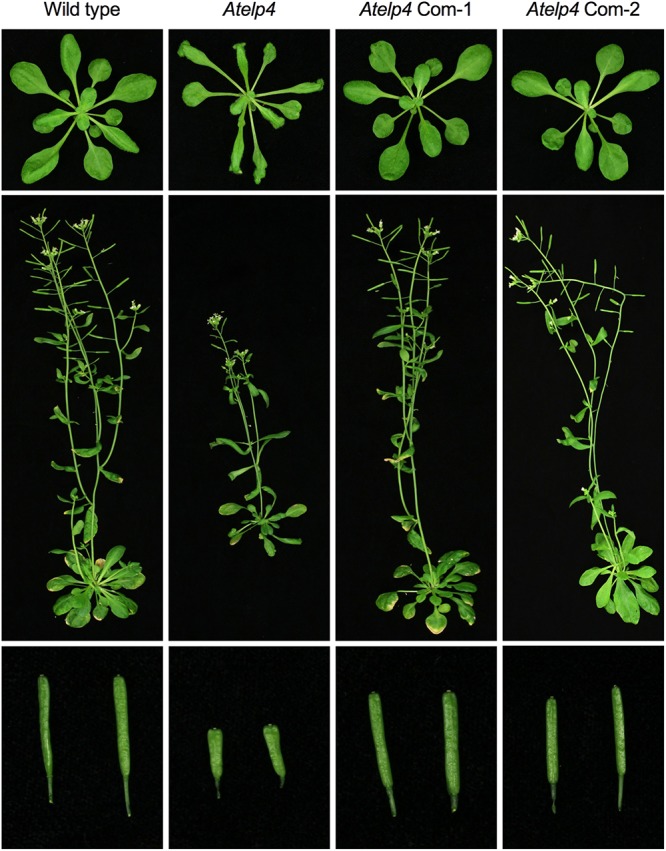
Complementation of the Arabidopsis *Atelp4* mutant by *SlELP4*. Morphological phenotypes of Arabidopsis wild type, *Atelp4*, and two independent complementation lines (*35S::SlELP4 Atelp4*): *Atelp4* Com-1 and *Atelp4* Com-2. **(Top)** 3-weak-old plants; **(Middle)** 6-week-old plants; **(Bottom)** siliques.

To assess the functionality of the SlELP3 and SlELP4 proteins, the *SlELP3* and *SlELP4* open reading frames driven by the 35S promoter were introduced into the *Atelp3* and *Atelp4* mutants, respectively, via *A. tumefaciens*-mediated genetic transformation. Multiple single insertion homozygous lines were obtained for both *SlELP3* and *SlELP4*. Morphologically, the *Atelp3* and *Atelp4* mutant plants have narrow leaves, long petioles, and shortened siliques (Nelissen et al., 2005). These morphological phenotypes all were completely restored to wild type in the transgenic *Atelp3* and *Atelp4* plants expressing *SlELP3* and *SlELP4*, respectively (**Figures 5, 6**). Thus, the functions of SlELP3 and SlELP4 are conserved.

**FIGURE 7:**
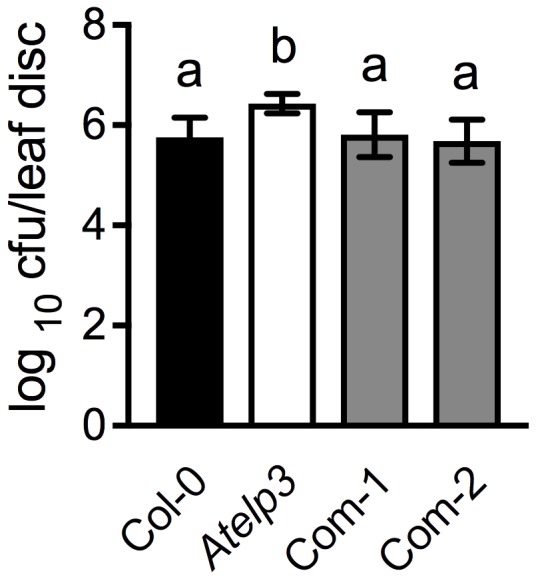
Complementation of the disease susceptibility phenotype of the *Atelp3* mutant by *SlELP3*. Four-week-old wild-type Col-0, *Atelp3*, and two independent complementation lines (Com-1 and Com-2) were inoculated with the bacterial pathogen *Psm* ES4326 (OD_600_ = 0.0001). The in planta bacterial titers were determined 3 days post-inoculation. Data represent the mean of eight independent samples with SD. Different letters above the bars indicate significant differences (Tukey’s test, *P* < 0.05).

To further confirm the morphological complementation results, we inoculated the wild-type Col-0, *Atelp3*, and two independent *Atelp3* complementation lines, Com-1 and Com-2, with the bacterial pathogen *P. syringae* pv. *maculicola* (*Psm*) ES4326 to test whether the enhanced disease susceptibility phenotype of *Atelp3* was also complemented by *SlELP3* (Defraia et al., 2013). As shown in **Figure 7**, while the *Atelp3* mutant was significantly more susceptible than the wild type to *Psm* ES4326, the growth of *Psm* ES4326 in the complementation lines was comparable to that in the wild type. This result indicates that the *SlELP3* gene can also complement the defense phenotype of the *Atelp3* mutant.

## Discussion

Bacterial speck, caused by *Pst*, is an important disease that concerns tomato growers worldwide (Bashan et al., 1978; Devash et al., 1980; Smitley and McCarter, 1982). Because of the lack of an effective control for the disease (Smitley and McCarter, 1982; Ramos et al., 1989), we investigated the potential of *Arabidopsis* defense-related genes for improvement of disease resistance against *Pst* J4 in tomato. The *Arabidopsis* genes encoding the Elongator subunits were chosen as the candidates for tomato transformation, as their effectiveness in mediating resistance to bacterial diseases has previously been demonstrated in *Arabidopsis* (Defraia et al., 2010, 2013; Wang et al., 2015; An et al., 2017) and strawberry (Silva et al., 2017). Additionally, given that Elongator functions at the chromatin level and is not directly involved in specific recognition of pathogens (Wang et al., 2013), the possibility of the pathogen’s ability to overcome the resistance is remote, which is critical for generating durable resistance in crop plants.

The aim of this work was to investigate the disease resistance of single insertion homozygous *AtELP3* and *AtELP4* transgenic tomato plants under growth chamber conditions. In total, we identified nine single insertion homozygous transgenic lines (**Figures 1A,B**). Although *Arabidopsis* Elongator mutants display striking morphological phenotypes (Nelissen et al., 2005), overexpression of *AtELP3* and *AtELP4* in tomato did not cause any abnormality. All transgenic lines except line 44-2 displayed morphological and developmental traits similar to those of the control (**Figures 1C–E**). The observed small-fruit phenotype of line 44-2 is not associated with the expression of the transgene *AtELP3* (**Figure 1A**), and may thus be caused by a T-DNA insertion mutation or seed contamination during transgenic plant development.

Overexpression of *AtELP3* and *AtELP4* both improved tomato resistance to bacterial speck, which is in agreement with the result reported in strawberry (Silva et al., 2017). However, the improvement conferred by *AtELP3* was marginal (**Figure 2B**). This result suggests that *AtELP3* may not be effective in improving disease resistance in tomato or that the AtELP3 protein levels accumulated in the five *AtELP3* transgenic lines used in this study may not be sufficient for activation of strong disease resistance. On the other hand, two *AtELP4* transgenic lines (23-1 and 61-5) displayed considerable enhanced resistance to bacterial speck under growth chamber conditions. The bacterial speck disease symptoms on both lines were dramatically alleviated (**Figures 2B–D**). Consistently, the bacterial populations in these transgenic lines were significantly lower over an 18-day period than those in the control (**Figure 2E**). We noticed that the enhanced disease resistance was not tightly correlated with the expression levels of the transgene, which is a common phenomenon in transgenic studies (Luhua et al., 2008). Unfortunately, no anti-AtELP4 antibody is available to determine the protein levels in the transgenic lines. Nevertheless, our results indicate that overexpression of *AtELP4* in tomato is able to significantly enhance resistance to *Pst* J4.

Interestingly, increased disease resistance was not observed when the bacterial suspension was infiltrated into the apoplast of the transgenic tomato plants (Supplementary Figure S1). This suggested that *AtELP3* and *AtELP4* might improve stomatal immunity in tomato. We therefore investigated if stomatal morphology and conductance were affected in the most resistant transgenic line. Surprisingly, inoculation of tomato plants with *Pst* J4 did not cause stomatal closure (Supplementary Figure S2), suggesting that *Pst* J4 has some mechanisms to keep stomata open (Cai et al., 2011). Moreover, no clear differences in stomatal morphology and conductance were detected between the transgenic line and the control (**Figure 3**; Supplementary Figure. S2). Further investigation is thus needed to understand the mechanisms underlying the observed resistance in the *AtELP3* and *AtELP4* transgenic lines, which is effective only when a spray inoculation method is used.

By using RNA-seq and qPCR, we identified a group of genes (*PR1b1, PR-5x, DES*, and *ER1*) that were induced to higher levels after *Pst* J4 infection in the most resistant *AtELP4* transgenic line than in the control (**Figure 4**). Note that these genes were not constitutively expressed in the transgenic tomato plants, which is different from the constitutive defense gene expression seen in the transgenic strawberry plants (Silva et al., 2017). These tomato genes have been reported to be involved in defense responses to pathogen infections. For instance, *PR1b1, PR-5x*, and *DES* have been shown to be associated with resistance to *Ralstonia solanacearum* in tomato (Ishihara et al., 2012). PR1b1 and PR-5x proteins were also found to accumulate in tomato xylem upon infection by *Fusarium oxysporum* (Rep et al., 2002), and divinyl ethers, the products of DES, have been reported to accumulate more rapidly in potato cultivars with increased resistance to late blight, a disease caused by *Phytophthora infestans* (Weber et al., 1999). Furthermore, The *ER1* gene has been shown to be associated with bacterial speck disease in tomato (Pautot et al., 1991). Therefore, the enhanced disease resistance in the *AtELP4* transgenic tomato plants is likely attributed to elevated induction of defense-related genes. Although it has been well demonstrated that Elongator regulates gene transcription by modifying chromatin structure (histone acetylation and/or DNA methylation) (Wang et al., 2013, 2015), whether overexpression of *AtELP4* in tomato alters the chromatin structure of the identified defense-related genes requires further investigation.

It is interesting that overexpression of a single Elongator subunit can dramatically improve tomato disease resistance. This phenomenon has also been seen in strawberry where overexpression of *AtELP3* or *AtELP4* significantly increased resistance to several bacterial and fungal pathogens (Silva et al., 2017). Such results appear to be in conflict with the notion that the Elongator complex functions as a whole and mutations in any of the subunits compromise the activity of the complex (Versées et al., 2010; An et al., 2016). However, it has been shown that overexpression of *ELP3*, but not *ELP4*, in human 293 T cells suppressed cell growth and enhanced transcription, and that overexpression of *ELP4* and *ELP3* together exhibited a synergistic effect on transcription activation (Gu et al., 2009). Moreover, elevating *ELP3* expression in yeast suppressed the *anaphase-promoting complex 5* mutant defects (Turner et al., 2010). These results together strongly suggest that individual Elongator subunits may either be able to increase the Elongator complex activity or have some Elongator complex-independent functions. Further investigation is clearly warranted to pinpoint the underlying molecular mechanisms.

Tomato appears to have a functionally conserved Elongator complex. The tomato genome contains single-copy genes encoding all SlELPs and the SlELP proteins share high identities and similarities with their corresponding AtELP proteins (Supplementary Figure S3). Furthermore, tomato orthologs of the *Arabidopsis AtELP3* and *AtELP4* genes, when transformed into the *Arabidopsis Atelp3* and *Atelp4* mutants, were able to restore wild-type morphology to the mutants (**Figures 5, 6**). Resistance to *Psm* ES4326 was also completely restored in the *Atelp3* mutant plants expressing the *SlELP3* gene (**Figure 7**). These results indicate that the functions of SlELP3 and SlELP4 are conserved and suggest that the function of the Elongator complex may be conserved in tomato. Indeed, silencing of *SlELP2* in tomato plants resulted in pleiotropic phenotypes similar to those of the *Atelp* mutants (Zhu et al., 2015). These results taken together indicate that the tomato Elongator complex not only is essential for plant fitness, but may also play an important roles in immunity. Although it has been shown that Elongator modulates the transcription of thousands of genes in *Arabidopsis* and *Saccharomyces cerevisiae* (Krogan and Greenblatt, 2001; Wang et al., 2013), how Elongator functions in tomato remains to be elucidated.

This study, together with our previous study (Silva et al., 2017), revealed several dramatic differences between the transgenic tomato and transgenic strawberry *AtELP3* and *AtELP4* plants. First, overexpression of *AtELP3* and *AtELP4* did not clearly impact tomato plant growth and development, which is in sharp contrast to the collateral effects observed in strawberry. Second, overexpression of *AtELP3* and *AtELP4* conferred resistance in tomato to bacterial speck caused by *Pst* J4 only when inoculated through foliar sprays but not through infiltration into the leaf apoplast, but in strawberry it provided resistance to the angular leaf spot-causing bacterial pathogen *Xanthomonas fragariae* when the pathogen was infiltrated into the apoplast. And finally, the elevated resistance in tomato is likely attributed to a stronger induction of defense responses in the transgenic plants than in the control, whereas in the transgenic strawberry plants resistance was associated with constitutive expression of defense genes. These results suggest that different plant species may respond differently to overexpression of genes encoding the subunits of the evolutionarily conserved Elongator complex. Further investigations are required to fully understand this interesting phenomenon.

## Author Contributions

JP, JJ, and ZM designed the research and wrote the manuscript. JP characterized transgenic plants and conducted qPCR assay. YZ performed RNA-seq analysis. FY analyzed RNA-seq data.

## Conflict of Interest Statement

ZM is a co-inventor on a patent application titled “Use of Elongator genes to improve plant disease resistance.”The remaining authors declare that the research was conducted in the absence of any other commercial or financial relationships that could be construed as a potential conflict of interest.

## Footnotes

1 www.arabidopsis.org

2 https://biotech.unl.edu/plant-transformation

3 https://www.licor.com

4 https://www.qiagen.com/

5 https://www.thermofisher.com/

6 https://www.agilent.com/

7 https://www.beckman.com

8 https://www.kapabiosystems.com/

9 http://www.appliedbiosystems.com/

10 https://www.genomics.agilent.com/

11 https://graphpad.com

12 https://solgenomics.net/organism/Solanum_lycopersicum/genome
