# Supplementary material for: The Arabidopsis Elongator Subunit ELP3 and ELP4 Confer Resistance to Bacterial Speck in Tomato
Source: Front Plant Sci. 2018 Jul 24;9:1066. doi: 10.3389/fpls.2018.01066 (PMC6066517; doi:10.3389/fpls.2018.01066)
Supplement: Supplementary file 1 [file Data_Sheet_1.DOCX]

**Supporting information**


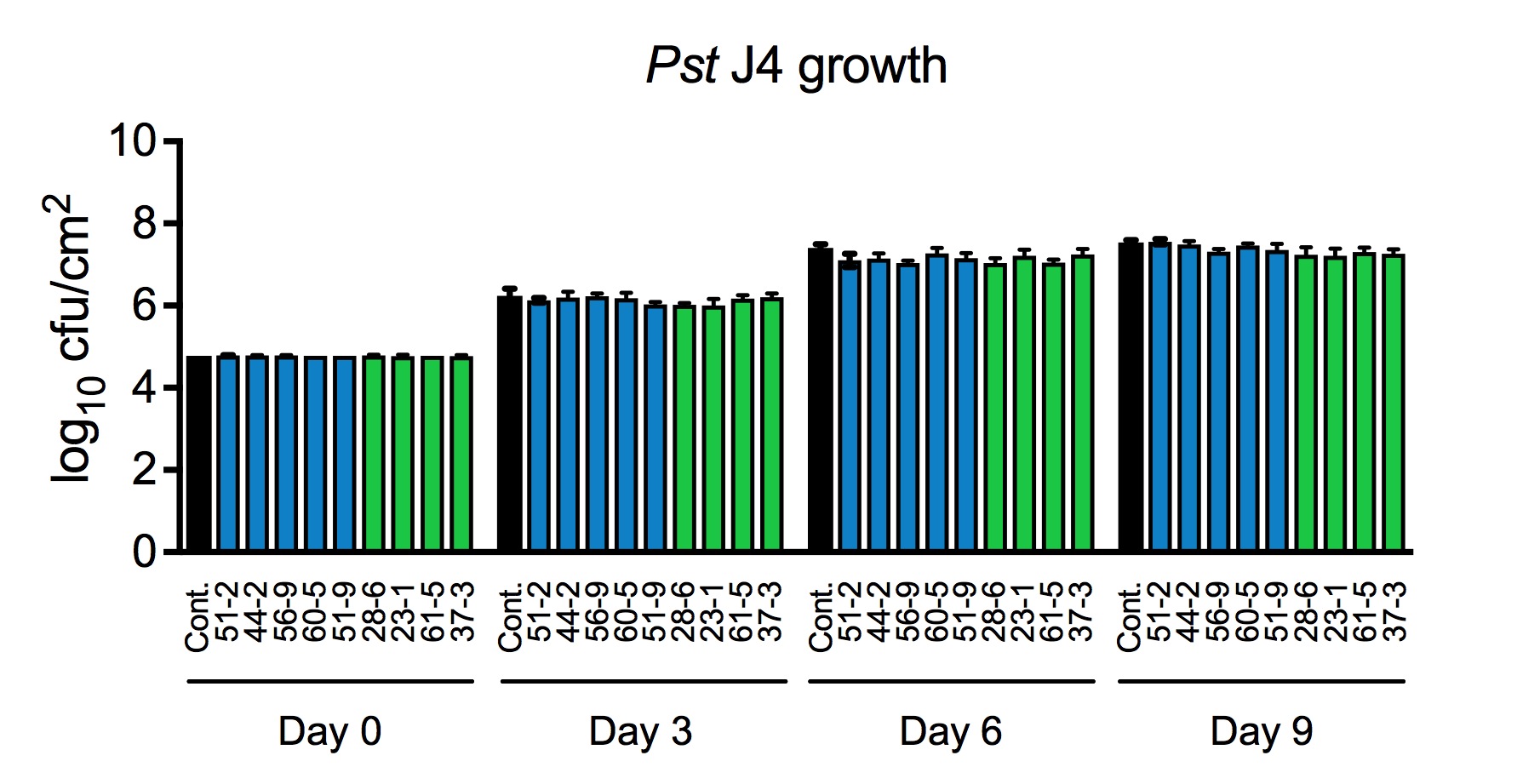


**Fig. S1** Bacterial titers in the transgenic lines and the control inoculated by leaf infiltration.

Leaves on four-week-old tomato plants were infiltrated with a suspension (1 × 10^5^ cfu/mL) of the bacterial pathogen *Pst* J4. cfu: colony forming unit; cont.: control. Data represent the mean of three biological replicates with standard deviation. No drastic differences were detected between *Pst* J4 growth in the transgenic line and that in the control.


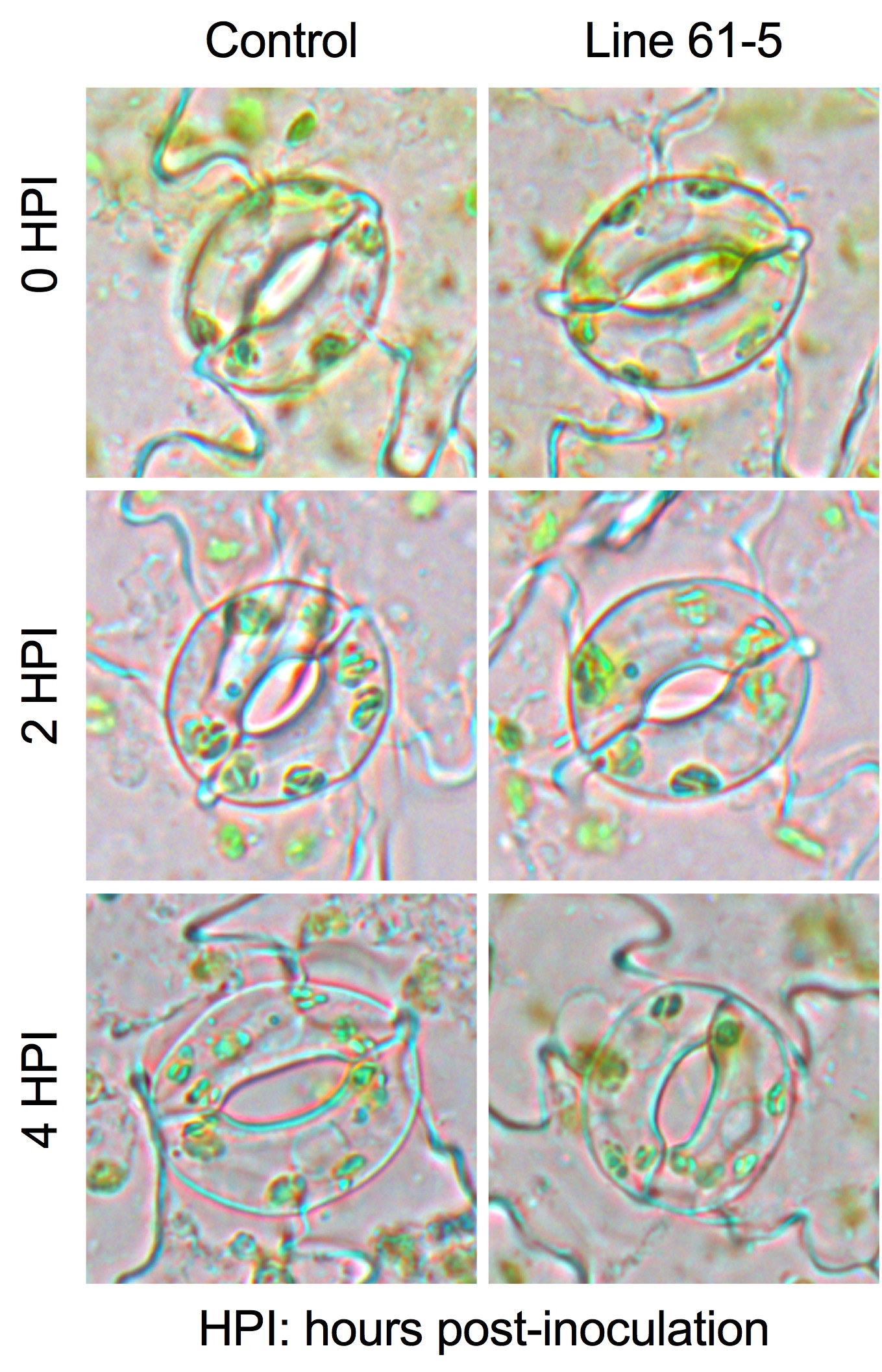


**Fig. S2** Morphology of stomata of the transgenic line 61-5 and the control.

Four-week-old tomato plants were sprayed with a suspension (10^8^ cfu/mL) of the bacterial pathogen *Pst* J4. Leaf tissues were collected at the indicated time points and epidermal peels were observed under optical microscope. No appreciable differences were observed between the stomata of the transgenic line and those of the control.

**Alignments between AtELP1 (length 1319) and SlELP1 (length=1314)**

Score = 1539 bits (3985), Expect = 0.0, Method: Compositional matrix adjust.

Identities = 780/1325 (59%), Positives = 982/1325 (74%), Gaps = 19/1325 (1%)

AtELP1 1 MKNLKLFSEVPQNIQLHSTEEVVQFAAYDIDQSRLFFASSANFVYALQLSSFQNESAGAK 60

MKNLK+ E IQL S +EV+ FAA+D++++RLF ASS+NF+Y L L S N AG

SlELP1 1 MKNLKILKEQFSKIQLQSEDEVISFAAFDVERNRLFLASSSNFIYTLSLPSSNN--AGGW 58

AtELP1 61 SAMPVEVCSIDIEPGDFITAFDYLAEKESLLIGTSHGLLLVHNVESDVTELVGNIEGGVK 120

+++ + +D+EPGDFIT+ DYL EKE+L+IGTS+GLLL++ + + TE+VG +EGGVK

SlELP1 59 NSISDNL--VDLEPGDFITSMDYLMEKEALIIGTSYGLLLLYTADDNTTEIVGRLEGGVK 116

AtELP1 121 CISPNPTGDLLGLITGLGQLLVMTYDWALMYEKALGEVPEGGYVRETNDLSVNCGGISIS 180

CISP+P GDLLG+ITG GQ+LVMT DW ++YE AL ++PE V E S IS

SlELP1 117 CISPSPDGDLLGVITGFGQILVMTPDWDVLYEMALDDLPEDIDVHEHTYSSNYSSESPIS 176

AtELP1 181 WRGDGKYFATMGEVYESGCMSKKIKIWESDSGALQSSSETKEFTQGILEWMPSGAKIAAV 240

WRGDGKYFAT+ V S + KK+KIWE DSGAL S SE+ F L+WMPSGAKIAAV

SlELP1 177 WRGDGKYFATLSRVNNSQTLHKKLKIWERDSGALHSVSESNSFMGSTLDWMPSGAKIAAV 236

AtELP1 241 YKRKSDDSSPSIAFFERNGLERSSFRIGEPEDATESCENLKWNSASDLLAGVVSCKTYDA 300

Y RK D PSI FFERNGLERSSF + DAT E +KWN SDLLA VV + YD+

SlELP1 237 YDRKEDRKCPSIVFFERNGLERSSFCLNVEIDAT--IELVKWNCNSDLLAAVVRGEKYDS 294

AtELP1 301 IRVWFFSNNHWYLKQEIRYPREAGVTVMWDPTKPLQLICWTLSGQVSVRHFMWVTAVMED 360

+++WF SNNHWYLKQEIRY ++ V MWDP KPLQL+ WT SG ++ +F+W TAVM +

SlELP1 295 LKIWFLSNNHWYLKQEIRYMKDDRVRFMWDPIKPLQLVTWTTSGHITGYNFVWNTAVMNN 354

AtELP1 361 STAFVIDNSKILVTPLSLSLMPPPMYLFSLSFSSAVRDIAYYSRNSKNCLAVFLSDGNLS 420

S A VID+SKIL+TPLSLSL+PPPMYLF L+F SA++ +A+ SR+S N LA LSDG L

SlELP1 355 SVALVIDDSKILITPLSLSLIPPPMYLFCLNFPSAIQSMAFCSRSSLNHLAASLSDGRLC 414

AtELP1 421 FVEFPAPNTWEDLEGKDFSVEISDCKTALGSFVHLLWLDVHSLLCVSAYGSSHNKCLSSG 480

VE PA + WE+LEGK+F V+ + + SF+HL WLD H LL VS Y S N +

SlELP1 415 VVELPAIDCWEELEGKEFDVDAASFDSGYNSFIHLAWLDSHKLLGVSHYLVS-NSAIKES 473

AtELP1 481 GYDTELHGSYLQEVEVVCHEDHVPDQVTCSGFKASITFQTLLESPVLALAWNPSKRDSAF 540

D +L LQE++++C ED +P+ VTCSG++A + LE V+ +A N SA+

SlELP1 474 SKD-KLSMYCLQEIDLMCSEDRLPNSVTCSGWQAKGLNRLSLEGTVIGIAPNQGNGCSAY 532

AtELP1 541 VEFEGGKVLGYASR-SEIMETRSSDDSVCFPSTCPWVRVAQVDASGVHKPLICGLDDMGR 599

V+F+GG+V YA + ++ + + F S+CPW+ + Q+ K L+ GLDD GR

SlELP1 533 VQFDGGEVFEYALKLADARGLHQKREDMSFSSSCPWMDLVQIGGCLPQKALLFGLDDSGR 592

AtELP1 600 LSINGKNLCNNCSSFSFYSELANEVVTHLIILTKQDFLFIVDTKDVLNGD--VALGNVFF 657

L + + LCNNCSSFSFYS A+ VTHLI+ TKQD LFIVD D+L G+ V GN F

SlELP1 593 LLVGERTLCNNCSSFSFYSNSADHSVTHLILSTKQDLLFIVDISDILKGELEVKYGN-FL 651

AtELP1 658 VIDGRRRDEENMSYVNIWERGAKVIGVLNGDEAAVILQTMRGNLECIYPRKLVLSSITNA 717

+ R+ E+ +Y+ IWERGA++IGVL+GDE+A+ILQT+RGNLEC+YPRKLVL+SI NA

SlELP1 652 AVFKHRKGEDERNYIQIWERGARIIGVLHGDESAIILQTVRGNLECVYPRKLVLASIINA 711

AtELP1 718 LAQQRFKDAFNLVRRHRIDFNVIVDLYGWQAFLQSAVAFVEQVNNLNHVTEFVCAMKNED 777

L Q R+KDA +VRR RIDFNVI+D GWQ F+QSA FV+QVNNL+++TEFVC++KNE+

SlELP1 712 LIQGRYKDALLMVRRQRIDFNVIIDHCGWQNFVQSAAEFVKQVNNLSYITEFVCSIKNEN 771

AtELP1 778 VTETLYKKFSFSKKGDEVFRV----KDSCSN-KVSSVLQAIRKALEEHIPESPSRELCIL 832

+ +TLYK + E V K S SN K+ SVL AIRKALEEH+ ESP+RELCIL

SlELP1 772 IMKTLYKNYISLPHDIEAKAVDGDLKSSHSNSKIHSVLLAIRKALEEHVTESPARELCIL 831

AtELP1 833 TTLARSDPPAIEESLLRIKSVREMELLNSSDDIRKKSCPSAEEALKHLLWLLDSEAVFEA 892

TTLARSDPPA+E++L RIK +RE EL + S ++R++ PSAEEALKHLLWL D+EAVFEA

SlELP1 832 TTLARSDPPALEQALERIKIIREREL-SGSGELRRELYPSAEEALKHLLWLSDTEAVFEA 890

AtELP1 893 ALGLYDLNLAAIVALNSQRDPKEFLPYLQELEKMPESLMHFKIDIKLQRFDSALRNIVSA 952

ALGLYDLNLAAIVALNSQ+DPKEFLPYLQELE MP LM + ID+KLQRF++AL++IVSA

SlELP1 891 ALGLYDLNLAAIVALNSQKDPKEFLPYLQELENMPIVLMRYNIDLKLQRFEAALQHIVSA 950

AtELP1 953 GVGYFPDCMNLIKKNPQLFPLGLLLITDPEKKLVVLEAWADHLIDEKRFEDAATTYLCCC 1012

G YF D M L+KKNPQLFP GL LITD K+ VLEAW DH K FEDAA TY+CC

SlELP1 951 GDAYFEDSMILMKKNPQLFPSGLQLITDSVKRNQVLEAWGDHFSSTKCFEDAAATYMCCS 1010

AtELP1 1013 KLEKASKAYRECGDWSGVLRVGALMKLGKDEILKLAYELCEEVNALGKPAEAAKIALEYC 1072

L+KA KAYRECG+W GVL V L+KLGK+E+L+LA ELC+E+ ALGKP +AAKIALEYC

SlELP1 1011 CLDKALKAYRECGNWGGVLTVAGLIKLGKEEVLQLAQELCDELQALGKPGDAAKIALEYC 1070

AtELP1 1073 SDISGGISLLINAREWEEALRVAFLHTADDRISVVKSSALECASGLVSEFKESIEKVGKY 1132

+D++ GI+ L++AREWEEALR AFL+ DD + VK+++LECAS LVSE++E +EKVGKY

SlELP1 1071 ADVNAGINFLVSAREWEEALRTAFLYRRDDLVLEVKTASLECASSLVSEYEEGLEKVGKY 1130

AtELP1 1133 LTRYLAVRQRRLLLAAKLKSEERSVVDLDDDTASEASSNLSGMSAYTLGTRRGSAASVSS 1192

LTRYL VRQRRLLLAAKL+S+ERS+ +LDDDTASE SSN SGMSAYTLGTR+GSAAS+ +

SlELP1 1131 LTRYLGVRQRRLLLAAKLQSDERSINELDDDTASETSSNFSGMSAYTLGTRKGSAASI-N 1189

AtELP1 1193 SNATSRARDLRRQRKSGKIRAGSAGEEMALVDHLKGMRMTDGGKRELKSLLICLVTLGEM 1252

S A+++ARD+RRQR GKIRAGS GEEM LV+HLKGM +T G KRELKSLLICLV L +

SlELP1 1190 SRASTKARDMRRQRNRGKIRAGSPGEEMGLVEHLKGMSLTSGAKRELKSLLICLVMLQKE 1249

AtELP1 1253 ESAQKLQQTAENFQVSQVAAVELAHDTVSSESVDEEVYCFERYAQKTRSTARDSDAFSWM 1312

+ A+KLQ A NFQ+SQ+AAV+LA + +S + V+E Y + Y K + + S+ FSW

SlELP1 1250 DIARKLQHVATNFQLSQMAAVKLADEAISDDIVNEHFYVLDNYIPKIKEDMQHSELFSWQ 1309

AtELP1 1313 LKVFI 1317

KV I

SlELP1 1310 SKVLI* 1314

**Alignments between AtELP2 (length 838) and SlELP2 (length=828)**

Score = 1108 bits (2866), Expect = 0.0, Method: Compositional matrix adjust.

Identities = 550/833 (66%), Positives = 668/833 (80%), Gaps = 18/833 (2%)

AtELP2 7 VEAKRVFIGAGCNRVVNNVSWGASGLVSFGAQNAVAVFCPKTAQILTTLPGHKASVNCTH 66

+E +RVFIGAGCNRVVNNVSWGASGLVSFGAQNAVA+FCPKTAQILTTL GHKASVNCT

SlELP2 4 MEVERVFIGAGCNRVVNNVSWGASGLVSFGAQNAVAIFCPKTAQILTTLAGHKASVNCTL 63

AtELP2 67 WLPTSKFAFKAKKLDRQYLLSGDSDGIIILWELSTLNNDWRHVLQLPLSHKKGVTCITAY 126

WLP SKFAFKAK+L++ LLSGD++G+IILWE S ++ WR+VLQ+P HKKGVTCITA

SlELP2 64 WLPNSKFAFKAKQLEQHLLLSGDAEGVIILWEYSLVDAKWRYVLQVPQVHKKGVTCITAI 123

AtELP2 127 MVSETDAMFASASSDGVVNVWDVSFPSQPSEECKVVCLDSICVDTKAIVTLSLAELPQNP 186

MVS+ +A+FASASSDG VNV +V FPS +CK+ C DS+ V K +V LSLAELP N

SlELP2 124 MVSQQEAVFASASSDGTVNVCEVVFPSTRGGDCKLSCSDSLFVGQKPMVALSLAELPGNS 183

AtELP2 187 GRFALALGGLDNKIKLYSGERTGKFTSVCELKGHTDWIRSLDFSLPLHTTEEIPNSIMLV 246

+ LA+GGLDNKI LY GER GKF CELK HTDWIRSLD SLP++ E +S++LV

SlELP2 184 KQLVLAMGGLDNKIHLYCGERNGKFLRACELKAHTDWIRSLDLSLPVYVNGE--SSLLLV 241

AtELP2 247 SSSQDKVIRIWKLVLVGDVGSWRREIT-LASYIEGPVFVSGTFTYQISVESVLIGHEDWV 305

SSSQDK IRIWK+ L S +++ T LASYI+GPV V+G+ +YQIS+ES+LIGHEDWV

SlELP2 242 SSSQDKGIRIWKMTLQDSSASNKKQQTSLASYIKGPVLVAGSSSYQISMESLLIGHEDWV 301

AtELP2 306 YSVEWQPPVIDFIDGRLVNHQPLSILSASMDKTMMIWRPEKKTGVWVNVVCVGELSHCAL 365

YSVEWQPP ++G + QP SILSASMDKTM+IW+PEK TG+W+NVV VGELSHCAL

SlELP2 302 YSVEWQPPSTSSVEG-IECFQPQSILSASMDKTMLIWQPEKTTGIWMNVVTVGELSHCAL 360

AtELP2 366 GFYGGHWSPNSLSILAHGYGGAFHLWRNVSSSKESENWQMQKVPSGHFAAVTDVTWARTG 425

GFYGGHWSPN+ ILAHGYGG+FHLW+NV E ++W+ QKVPSGHFAAV+D+ WAR G

SlELP2 361 GFYGGHWSPNADFILAHGYGGSFHLWKNV--GIEYDDWKPQKVPSGHFAAVSDIAWARCG 418

AtELP2 426 EYLLSVSQDQTTRVFSAWKNDEGNEAEDEHWHELARPQVHGHDINCVAMVQGKGNHRFVS 485

EY++SVS DQTTRVF+ W N+ + E E WHE+ARPQVHGHDINCV +++GKGNHRFV

SlELP2 419 EYMMSVSHDQTTRVFAPWLNNTSVQNE-ESWHEIARPQVHGHDINCVTVIKGKGNHRFVG 477

AtELP2 486 GAEEKVVRVFEAPLSFLKTLNHTCAGGEGSFPEDLQADVQVLGANMSALGLSQKPIYLHS 545

GA+EKV RVFE+PLSFLKTL+H + SF D+QADVQ+LGANMSALGLSQKPIY+ +

SlELP2 478 GADEKVARVFESPLSFLKTLSHVTS-DNSSFSADIQADVQILGANMSALGLSQKPIYVQA 536

AtELP2 546 SSEPLERNGGGEGLDTFETVPEAAPAELKEPPIEDQLAFHTLWPESHKLYGHGNELFSLC 605

S+ P++R+ EG DT ETVPEA P L EPPIE+QLA+HTLWPESHKLYGHGNELFSLC

SlELP2 537 ST-PIDRS-NTEGFDTLETVPEAVPVVLTEPPIEEQLAWHTLWPESHKLYGHGNELFSLC 594

AtELP2 606 SDHKGNLVASSCKAQSASMAEIWLWEVGTWKAVGRLQSHSLTVTHLEFSYDDTLLLSVSR 665

DH G LVASSCKAQSA +AEIWLW+VG+WK+VGRL+SHSLTVT +EFS+D+ LL+VSR

SlELP2 595 CDHDGKLVASSCKAQSAPVAEIWLWQVGSWKSVGRLRSHSLTVTQMEFSHDNKYLLAVSR 654

AtELP2 666 DRHFSVFSIQRTDNGEVSHKLMAKVEAHKRIIWACSWNPFGHQFATSSRDKTVKIWSVEN 725

DRHFSVF I E+ ++L+AK EAHKRIIW+CSWNPFGH+FAT SRDKTVKIW+V

SlELP2 655 DRHFSVFQINHKGTDEIDYQLVAKQEAHKRIIWSCSWNPFGHEFATGSRDKTVKIWAVGT 714

AtELP2 726 DARIKQILVLPPFGSSVTAVAWTGLDRNEKSGCVAVGMESGLIELSNVKIIETEEG---- 781

+ +K +L LPPF SSVTA++W LD + G +AVGME+GLIEL N +++ G

SlELP2 715 ETSVKLLLTLPPFKSSVTALSWLSLDNHSNHGLLAVGMENGLIELWN---LDSRGGDGHL 771

AtELP2 782 TTATAALALRLEPFMCHVSAVNRLAWRPTEKCESNQSLRWLTSCGDDNCVRVF 834

+ A+ A++ +PF+CHVS V RL+WR +K E +++++ L SCG D+CVR+F

SlELP2 772 SVQNASPAVKFDPFLCHVSTVQRLSWRNPQKSEDSETVQ-LASCGADHCVRIF 823

**Alignments between AtELP3 (length 565) and SlELP3 (length=565)**

Score = 1083 bits (2802), Expect = 0.0, Method: Compositional matrix adjust.

Identities = 518/557 (93%), Positives = 539/557 (96%), Gaps = 0/557 (0%)

AtELP3 10 ELKKQPRPGKGGYQGRGLTEEEARVRAISEIVSTMIERSHRNENVDLNAIKTAACRKYGL 69

E +K PRPG+GG GLTEEEARVRAI+EIV+ M+E S + ++VDLNA+K+AACRKYGL

SlELP3 10 ETRKLPRPGRGGVVSLGLTEEEARVRAITEIVNNMVELSRKGKDVDLNALKSAACRKYGL 69

AtELP3 70 ARAPKLVEMIAALPDSERETLLPKLRAKPVRTASGIAVVAVMSKPHRCPHIATTGNICVY 129

+RAPKLVEMIAALPDSERETLLPKLRAKPVRTASGIAVVAVMSKPHRCPHIATTGNICVY

SlELP3 70 SRAPKLVEMIAALPDSERETLLPKLRAKPVRTASGIAVVAVMSKPHRCPHIATTGNICVY 129

AtELP3 130 CPGGPDSDFEYSTQSYTGYEPTSMRAIRARYNPYVQARSRIDQLKRLGHSVDKVEFILMG 189

CPGGPDSDFEYSTQSYTGYEPTSMRAIRARYNPYVQARSRIDQLKRLGHSVDKVEFILMG

SlELP3 130 CPGGPDSDFEYSTQSYTGYEPTSMRAIRARYNPYVQARSRIDQLKRLGHSVDKVEFILMG 189

AtELP3 190 GTFMSLPAEYRDFFIRNLHDALSGHTSANVEEAVAYSEHSATKCIGMTIETRPDYCLGPH 249

GTFMSLPAEYRD+F RNLHDALSGHTSANVEEAVAYSEH ATKCIGMTIETRPDYCLGPH

SlELP3 190 GTFMSLPAEYRDYFTRNLHDALSGHTSANVEEAVAYSEHGATKCIGMTIETRPDYCLGPH 249

AtELP3 250 LRQMLIYGCTRLEIGVQSTYEDVARDTNRGHTVAAVADCFCLAKDAGFKVVAHMMPDLPN 309

LRQML YGCTRLEIGVQSTYEDVARDTNRGHTVAAVADCFCLAKDAGFKVVAHMMPDLPN

SlELP3 250 LRQMLSYGCTRLEIGVQSTYEDVARDTNRGHTVAAVADCFCLAKDAGFKVVAHMMPDLPN 309

AtELP3 310 VGVERDMESFKEFFESPSFRADGLKIYPTLVIRGTGLYELWKTGRYRNYPPEQLVDIVAR 369

VGVERD+ESFKEFFESPSFR DGLKIYPTLVIRGTGLYELWKTGRYRNYPPEQLVDIVAR

SlELP3 310 VGVERDLESFKEFFESPSFRTDGLKIYPTLVIRGTGLYELWKTGRYRNYPPEQLVDIVAR 369

AtELP3 370 ILSMVPPWTRVYRVQRDIPMPLVTSGVEKGNLRELALARMDDLGLKCRDVRTREAGIQDI 429

ILSMVPPWTRVYRVQRDIPMPLVTSGVEKGNLRELALARMDDLGLKCRDVRTREAGIQDI

SlELP3 370 ILSMVPPWTRVYRVQRDIPMPLVTSGVEKGNLRELALARMDDLGLKCRDVRTREAGIQDI 429

AtELP3 430 HHKIKPEQVELVRRDYTANEGWETFLSYEDTRQDILVGLLRLRKCGKNVTCPELMGKCSV 489

H+KI+PE+VELVRRDYTANEGWETFLSYEDTRQDILVGLLRLRKCG+NVTCPEL G+CS+

SlELP3 430 HNKIRPEEVELVRRDYTANEGWETFLSYEDTRQDILVGLLRLRKCGRNVTCPELTGRCSI 489

AtELP3 490 VRELHVYGTAVPVHGRDADKLQHQGYGTLLMEEAERIARREHRSNKIGVISGVGTRHYYR 549

VRELHVYGTAVPVHGRD DKLQHQGYGTLLMEEAERIARREHRS KI VISGVGTRHYYR

SlELP3 490 VRELHVYGTAVPVHGRDTDKLQHQGYGTLLMEEAERIARREHRSTKIAVISGVGTRHYYR 549

AtELP3 550 KLGYELEGPYMVKHLL* 566

KLGYELEGPYMVK+L+*

SlELP3 550 KLGYELEGPYMVKNLV* 566

**Alignments between AtELP4 (length 355) and SlELP4 (length=360)**

Score = 445 bits (1145), Expect = 7e-166, Method: Compositional matrix adjust.

Identities = 228/352 (65%), Positives = 280/352 (79%), Gaps = 6/352 (1%)

AtELP4 11 SFSRNISVVSSPQIPGLKSGPNGTAFISSGIRDLDRILGGGYPLGSLVMVMEDPEAPHHM 70

SFSRNIS ++ QIPG+K GPNGT+F+SSGI DLD+ILGGG+ LGSLVMVMEDPEAPHHM

SlELP4 10 SFSRNISSAATAQIPGVKLGPNGTSFLSSGIPDLDQILGGGFTLGSLVMVMEDPEAPHHM 69

AtELP4 71 DLLRTYMSQGLVNNQPLLYASPSKDPKGFLGTLPHPASSKEDKPTAPDPDQGESLRIAWQ 130

LLR +MSQGL++ QPLLYASP +DP+GFLGTLP P +SKE+K +Q +LRIAWQ

SlELP4 70 LLLRNFMSQGLIHKQPLLYASPERDPRGFLGTLPSPMASKEEKSNERPSEQDANLRIAWQ 129

AtELP4 131 YRKYL----ENQKNAIDDYSNDFDMRKPLERQFLSGRPIDCVSLLDSSDLSIAQDHCATF 186

Y+KY E Q+ +Y NDFD+RKPLER F SG+ +DC+SL DS +L + C+TF

SlELP4 130 YKKYFGEQTEVQRGGKAEYCNDFDLRKPLERHFYSGQRVDCISLRDSPNLVPLLERCSTF 189

AtELP4 187 LSKFPRNSSNIASIGRIAIQSFCSPLCEYSEKESDMLSFIRLLKSMLMVSNAVAIVTFPP 246

++ ++ NI GRIAIQS CSP C++S+K+ +MLSFIR LK M+ S AVA+++FPP

SlELP4 190 SAQISKSDGNITCAGRIAIQSLCSPQCDFSDKDWEMLSFIRSLKGMVRSSGAVAVISFPP 249

AtELP4 247 SLLSPSSSKRLQHMADTLLSIKAIPDGDKELEKLLTGYKDINGFLNIHKVARINTQVPVI 306

SL+SP+ KR QH+ADTL+S+KAIPD DKEL KLLTGY+D+ G L++HKVARINTQVP I

SlELP4 250 SLVSPAFLKRWQHLADTLISVKAIPDEDKELAKLLTGYQDMLGLLSVHKVARINTQVPAI 309

AtELP4 307 LEAKTFSMSLKKRRFLALECLNQAPVDGSSGTSYGTSGSC--SSKSGALDF* 356

LEA TFSM L+KRR L LECLNQAPVDGSSG+SYGTSG+C SSK+G LDF*

SlELP4 310 LEATTFSMKLRKRRALVLECLNQAPVDGSSGSSYGTSGACSGSSKTGNLDF* 361

**Alignments between AtELP5 (length 374) and SlELP5 (length=376)**

Score = 388 bits (996), Expect = 1e-121, Method: Compositional matrix adjust.

Identities = 202/368 (55%), Positives = 268/368 (72%), Gaps = 10/368 (2%)

AtELP5 1 MAESIFRKLRDGGEEGELAPALTIEETVASPFGLDVSGYLLTNLSSSILAGKSSSQGLVL 60

MAE+I R LRDG EGE APALTI++T+ +P G V ++LT L+S+ILAGKS ++G+VL

SlELP5 1 MAETICRALRDGAFEGEHAPALTIKDTIDAPLGSFVFNHILTQLTSNILAGKSQARGVVL 60

AtELP5 61 ITFSRSPSFYLQLLKQKGIVVSSSSKWIRILDCYTDPLGW----IDQSSTSFSEGSSLIK 116

+ SR PSFY++LLK KG VSSSSKW+R+LDCY+DPLGW +++ + +L+K

SlELP5 61 VALSRPPSFYVELLKNKGFDVSSSSKWLRVLDCYSDPLGWKNKLLERGTVRNPYEETLLK 120

AtELP5 117 LHKC--VSDLKKLFSSIIEAGRELVGTGKTRFCVAIDSVNELLRHSAMPLVSGLLTDLRS 174

C + +L K+ SSIIE G+E+V GK RF VAIDSV+E+LRHS++P V+ +L+ LRS

SlELP5 121 TTLCKNLKELDKVLSSIIELGKEIVEEGKGRFAVAIDSVSEILRHSSLPSVARILSHLRS 180

AtELP5 175 HAQISSVFWSLNTDLHQEKVTNALEYISTMKANLEPLCPSSDGQRNALENLFSVHQDFGK 234

H Q+S +F L+ DLH+ KV LEY+STM A++EP+ ++GQRN E+L V Q F +

SlELP5 181 HDQVSCIFCLLHVDLHEAKVAATLEYLSTMHADVEPIVQRTNGQRNTSEDLPMVEQSFKR 240

AtELP5 235 GRFHVRFKLRKGRVRVMSEEYHVDQSGINFSPISSVDTVIAATKSLLPKVQFNLQLSEKE 294

G+FHVRFK R GRVRVM EE +V+ SGI + +SS D + A +SL+PKVQFNL+LSEKE

SlELP5 241 GKFHVRFKRRNGRVRVMREELYVEGSGIKITEVSSEDGITA--QSLVPKVQFNLELSEKE 298

AtELP5 295 RVEKEKVVLPFEHQDDGKSNEIYDGRRSLVDGKIETTPLSSMELQTDVVSSGKGGEIIYF 354

R+++ KVVLPFEHQ GK +IYDGR+SL + + E S +LQT SG+ GEIIYF

SlELP5 299 RLDRAKVVLPFEHQGTGKPIQIYDGRKSLNESENEEKQASVEKLQT-TEDSGR-GEIIYF 356

AtELP5 355 RDSDDEHP 362

RDSDDE P

SlELP5 357 RDSDDEMP 364

**Alignments between AtELP6 (length 262) and SlELP6 (length=254)**

Score = 276 bits (705), Expect = 2e-100, Method: Compositional matrix adjust.

Identities = 140/264 (53%), Positives = 187/264 (70%), Gaps = 12/264 (4%)

AtELP6 1 MDRS-LNLLDLALGFDEQLAIPSPLNGKVILIEDCVETSGSFVLHQLMKRVLSSNSSDAL 59

MD S NLL+ A+G ++ G+V+ +EDCVETSG+FVL+ +KR L +SSD +

SlELP6 1 MDNSRANLLEEAVGINK--------GGRVVAVEDCVETSGAFVLYHFLKRSLHPDSSDVV 52

AtELP6 60 IFLAFARPFSHYDRILRKLGCNLATHKSNNRLVFFDMLMVKCSDGDQMEDNVSAVAKLFR 119

IF+AF+ PFSHY+RILRK+GCNL H+ N+R VF DML ++C D + E + L+

SlELP6 53 IFIAFSHPFSHYERILRKMGCNLTVHRKNHRFVFLDMLTLECPDRNGKERRQDGLLALYG 112

AtELP6 120 EIQETVRKLQSVT-SGNITVMVDDMSLLEIATTGSNSDHVLDFLHYCHTLSSESNCSLVI 178

EI++ V S+ S IT+M+DD+SL+E+A GS S+HVLDFLHYC+TL ++ CS V

SlELP6 113 EIEKAVEIYSSLEGSRTITIMIDDVSLIEVAANGS-SNHVLDFLHYCYTLKAKYGCSFVT 171

AtELP6 179 LNHEDIYASMERPAFLLQMVCLADVVIKAEPLASGLANDVHGQLTVLNKGISNSGRGSSR 238

LNHEDIY+S +LQ ADV+IKAEPLA+GLA+DVHGQLTVLNKG S G S

SlELP6 172 LNHEDIYSSANMLPLILQPEYFADVIIKAEPLATGLASDVHGQLTVLNKG-SVCDLGGSS 230

AtELP6 239 NKLQNFQFRIKENGIDYFYPGCRS* 262

+K++NF FR+KEN +DYFYPG ++*

SlELP6 231 SKVRNFHFRVKENIVDYFYPGTQT* 254

**Fig. S3** Amino acid alignments between AtELPs and SlELPs.

The amino acid sequences of AtELPs and SlELPs were entered into the online Align Sequences Protein BLAST tool (https://blast.ncbi.nlm.nih.gov/Blast.cgi?PAGE=Proteins&PROGRAM=blastp&BLAST_PROGRAMS=blastp&PAGE_TYPE=BlastSearch&BLAST_SPEC=blast2seq&DATABASE=n/a&QUERY=&SUBJECTS=) and the alignment results are presented.

**Table S1** Primers used in this study.

| **Primers** | **Sequences (5’ to 3’)** |
| --- | --- |
| Cloning | |
| *XhoI-SlELP3F* | CCGCTCGAGATGGCGGCGGCGGCGGTAGC |
| *SacI_SlELP3R* | CGAGCTCTACACAAGGTTTTTTACCATGTAAG |
| *SalI-SlELP4F* | ACGCGTCGACATGGCTTCAAGTAGACCGCG |
| *SacI-SlELP4R* | CGAGCTCTAGAAGTCGAGGTTCCCGG |
| Genotyping | |
| *35S-F* (for *AtELP3* lines) | ACCACGTCTTCAAAGCAAGTG |
| *AtELP3R* | GCGGTCGACTCAAAGAAGATGCTTCACCATGTAAG |
| *AtELP4F* | AAGAGGATAAGCCTACTGCG |
| *AtELP4R* | TCCGGATTTGGATGAGCAGC |
| qPCR | |
| *AtELP3F* | ACACTGGATATGAGCCTACC |
| *AtELP3R* | ACTCAGCAGGCAGTGACATG |
| *AtELP4F* | ATGGAAGATCCTGAAGCACC |
| *AtELP4R* | CTTTGGATGATGCAGGATGC |
| *PR1b1F* | GGTCGGGCACGTTGCA |
| *PR1b1R* | GATCCAGTTGCCTACAGGACATA |
| *PR-5xF* | GCCATGCAATTCATTGCA |
| *PR-5xR* | ATTGTTGTCCTCCGAACG |
| *DES-F* | ATGGTTCAAAGCCACGAT |
| *DES-R* | TCTCACCATCATTCATGAACC |
| *ER1F* | GCTTTCAATCTCTCACTGCTC |
| *ER1R* | CTTAGCTGGTGTTCCAAGAAG |
| *ActinF* | TTGCCGCATGCCATTCT |
| *ActinR* | TCGGTGAGGATATTCATCAGGTT |
